# Fidelity assessment of the suicide reporting guidelines in Korean newspapers

**DOI:** 10.1186/s12889-018-6014-4

**Published:** 2018-09-12

**Authors:** JongSerl Chun, Jinyung Kim, Serim Lee

**Affiliations:** Department of Social Welfare, Ewha Womans University, 52, Ewhayeodae-gil, Seodaemun-gu, Seoul, 03760 Republic of Korea

**Keywords:** Suicide, Reporting guidelines, Newspapers, South Korea

## Abstract

**Background:**

With the dishonor of being the highest suicide rated country in Organisation for Economic Co-operation and Development, South Korea should take more initiatives in suicide prevention. Although the role of the media and its relation to actual suicide attempts has been tested and supported by many studies, the suicide reporting guidelines are not well followed. The purpose of this study is to examine how well Korean newspapers adhere to existing guidelines and to suggest limitation and improvements for the current guidelines.

**Methods:**

Five mainstream newspapers in South Korea, namely, *Kyunghyang Shinmun*, *Hankyoreh*, *Chosun Ilbo*, *JoongAng Daily*, and *Dong-A Ilbo*, were chosen for the analysis. Using the Naver news search engine, articles dated from January 1, 2014, to December 31, 2017, were selected with the keyword “suicide” and advanced option “printed newspaper.” However, articles, columns, opinions, and reviews that utilized the word “suicide” in a general context were excluded from the final analysis. Finally, the number of cases was narrowed down to 368. Each article was analyzed using the guideline framework consisting of 13 items: sensational coverage, overstatement, direct wording, method used, details about site/location, photographs, suicide note, generalization, speculation, romanticization, interviews with the bereaved, help-seeking information, and public education.

**Results:**

More than 60% of the articles included direct wording (63.9%), mentioned the method used (68.2%), and provided details about the site or location (74.5%). Nearly half of the articles revealed the contents of the suicide note (44.6%). Less than 3% of the suicide reports had information about hotline logo or phone numbers (1.4%) and facts regarding suicide and suicide prevention (2.2%).

**Conclusions:**

Our study revealed that the guidelines were ineffective in their monitoring role and that most of the newspapers were incompliant with many significant guideline items in South Korea. Our findings not only explore the limitations of the current guidelines but also provide an important rationale as to why there should be stronger suicide monitoring regulation or an agency with sufficient authority to prevent suicide in a nation-wide scale.

## Background

According to World Health Organization (WHO) suicide data [1], nearly 800,000 people die of suicide every year. Suicide not only accounts for 1.4% of all deaths worldwide but is also the second leading cause of death among 15–29-year-olds globally. Of the Organisation for Economic Co-operation and Development countries, South Korea has by far the highest suicide rate. While South Korea’s suicide rate reached 26.5% per 100,000 people in 2015, the country with the second highest suicide rate, Hungary, had a rate of 19.4% in the same year [2, 3]. Suicide is especially high among the elderly population and it is the first cause of death among 10–30-year-olds in South Korea [4].

Based on several theoretical studies, it has been found that the frequency with which a person is exposed to suicide coverage can have a significant impact on his/her making an actual suicide attempt [5–9]. For example, in social learning theory, the greater the similarity between the model and the learner, the more likely it is that the learner will imitate the model’s behavior; this is labeled as “copycat suicide” [5]. Modeling theory suggests that a particular pattern of behavior described in the media can become a model for the audience [6]. Thom et al. [9] suggest that when the media covers suicide stories with detailed methods, visual items, glamorization, emphasis on celebrities, or sensationalism, the number of imitation suicides surges and the audience starts to perceive suicide as a reasonable solution for life challenges.

Suicide reported cases on media is unknowingly pervading into our daily lives, thoughts, and action. According to the Korea Association for Suicide Prevention, following the two-month period after the suicide of a Korean actress in 2008, the number of people committing suicide increased by 41.4% (2007 = 1807/2008 = 3081) because of the Werther effect [10]. Werther effect refers to an increase in the number of suicides in a population triggered by media reporting on a suicide case [11]. Similarly, the number of railway suicidal acts increased by 18.8% compared to the 2-year period before the railway suicide of a famous German goalkeeper [11].

Since people attempt to commit suicide or feel sympathy toward the person involved in the suicidal act [12–14], media should feel more responsible to abide by suicide reporting guidelines than any others. Gould et al. [8] proposed that the amount, duration, and prominence of media coverage are proportionate to an increase in suicide rates. Newspapers were more influential medium causing copycat suicides than television by 82% [15]. This is because, unlike television, which sends out visualized news coverage in a short time, newspapers can convey more details to the public without a time limit [15]. Newspaper articles can be easily accessed, saved, and reread through various media as well thus it enables the readers to regurgitate the materials [16]. Additionally, unlike printed newspapers, online articles can be reproduced continuously and without limit, exposing the readers more frequently to suicide contents [17].

Given the amount of research conducted in this field, suicide reporting guidelines should have a considerable impact on preventing suicide. Acknowledging such importance, a number of international and nationwide organizations, such as the WHO, the Centers for Disease Control and Prevention (CDC), Samaritans, Mindset, and Korean Ministry of Health and Welfare have established their own suicide reporting guidelines [18–22].

The WHO launched its first guidelines for suicide prevention in 1999 and the current guidelines are the second set to address specific social and professional groups for preventing suicide [21]. The CDC suicide guidelines are based on more than 50 international studies on suicide contagion and were initially developed by the American Foundation for Suicide Prevention and several leading experts in suicide prevention, along with several public and media organizations [18]. Samaritans produced the UK media guidelines following extensive consultation with journalists and editors throughout the industry [19]. Its guideline provides practical recommendations for reporting on suicide across all types of media to support high-quality journalism and help reporters avoid common pitfalls [19]. Mindset’s guidelines are commonly used in Canada; however, its work is the result of collaboration between the American Association of Suicidology and the Canadian Association for Suicide Prevention [20]. Mindset’s guideline consists of general statements to aid in the responsible presentation of information about suicide [20].

In Korea, the first suicide reporting guidelines were announced by the Korean Ministry of Health and Welfare in 2004, and they were updated on September 9, 2013, with three additional principles and one revised [22]. Principles such as respecting the bereaved family, not mentioning the details of the suicide, not romanticizing the act, and avoiding sensationalized expression remained the same in the updated version. However, more specific and preventive measures, such as principles 2 (usage of direct wording), 7 (information regarding consequences), 8 (conveying accurate information about suicide prevention), and 9 (paying special attention to online reports) have been added in the new guidelines (Table 1).

**Table 1:** Principles of “Suicide Reporting Guideline 2.0”

Nine Principles of the “Suicide Reporting Guideline 2.0” (9/9/2013)	
1. Minimize reports on suicide 2. Refrain from using the word “suicide” and avoid sensational expressions 3. Minimize detailed information about the suicide 4. Carefully consider the feelings of the bereaved family and accord them respect 5. Avoid romanticization or making statements about suicide or those who commit suicide 6. Should not utilize the suicide report to question social issues 7. Inform the public about the consequences of suicide 8. Provide accurate information about suicide prevention 9. Pay special attention when reporting suicide online	

Source: Korean Ministry of Health and Welfare [22]

The levels of concordance and discordance of media reports with suicide guidelines can provide important implications for further research and the improvement of the current guidelines. Creed and Whitley [23] found that 55% of the Canadian newspapers that reported on the suicide of a famous figure had adhered to Mindset’s guidelines by up to 80%. In the study of Ramadas and Kuttichira [24], they referred to the WHO guidelines and the suggestions of professionals in the related field to create their analytical framework. They observed an increase in the percentage of guideline concordance following the development of suicide reporting guidelines in India. Harshe et al. utilized the guidelines formulated by the Indian Psychiatric Society [25]. Having to see the increasing numbers of detailed news stories after a celebrity suicide, they expressed concern over the possible spurts of copycat suicide in India. In the United Kingdom, the online media was poorly compliant with Samaritans’ guidelines in reporting suicide cases [26]. As a result, researchers of this study saw the need to implement responsible and clear guidelines that could improve the poor quality of online media reporting and to train journalists appropriately.

However, unlike other countries, South Korea is significantly lacking in the study of suicide reporting guidelines. Kim [27] analyzed the difference in the number of suicide reports before and after the implementation of “Suicide Reporting Guideline 1.0” in 2004. Only two items were statistically significant, making it hard to conclude that the intervention was effective. Yeo and Lee [28] highlighted problems and suggested solutions for the existing suicide reporting guidelines; however, their study only focused on the coverage of specific celebrity suicides in three Korean broadcasting channels. In addition, Yu [29] carried out an exploratory study for establishing more realistic media guidelines for the reporting of suicides. Rather than analyzing newspaper articles, the study carried out a survey asking what factors should be included in the guidelines.

While the importance of developing suicide guidelines continuously rises, only a few studies have explored their fidelity. Therefore, the purpose of this study is to investigate how well Korean newspapers follow suicide reporting guidelines and to explore the potential problems and limitations regarding the current guidelines.

## Methods

### Sample

**Fig. 1:**
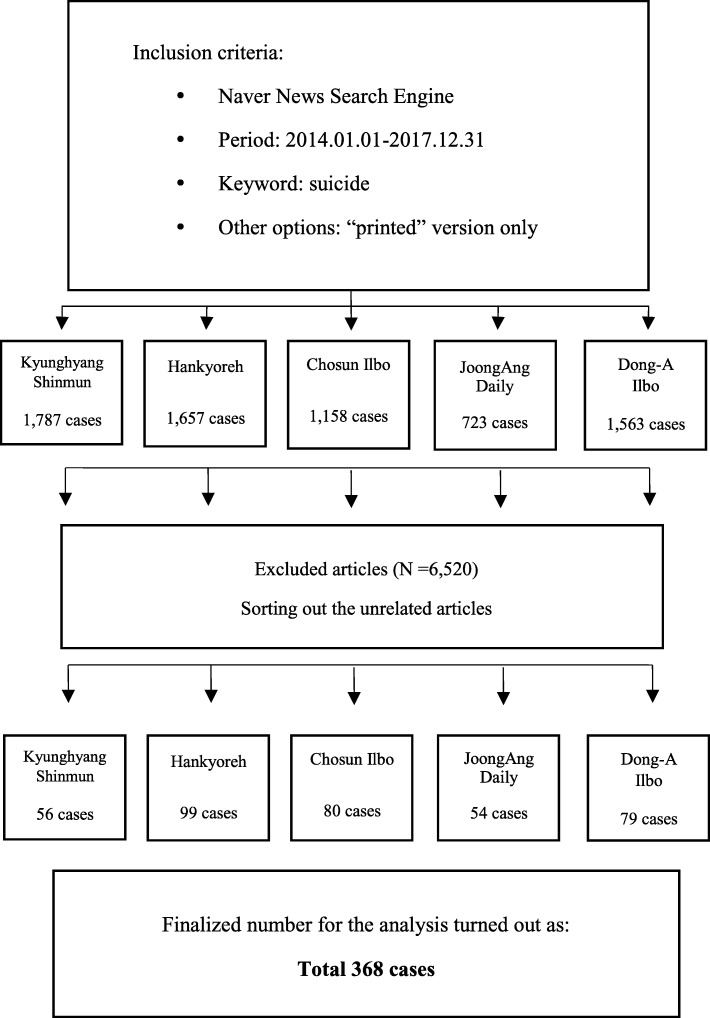
Sampling Procedure

Five mainstream newspapers in South Korea, namely*, Kyunghyang Shinmun*, *Hankyoreh, Chosun Ilbo*, *JoongAng Daily*, and *Dong-A Ilbo*, were chosen since they are commonly known and read among the Korean public. Through Naver news search engine, articles dated from January 1, 2014, to December 31, 2017, were selected with the keyword “suicide” and the advanced option of “printed newspaper.” It yielded a total of 6888 cases. However, articles, columns, opinions, and reviews that utilized the word “suicide” in a general context were excluded from the final analysis. In the end, the number of cases was narrowed down to 368 (Fig. 1).

### Analysis

A combination of several guidelines including those proposed by the WHO, CDC, Samaritans, and Mindset was used to create a framework for the analysis in this study [18–21]. Each article was coded for either presence/yes (1) or absence/no (0) of the guideline items shown in Table 2. Prior to the coding procedure, two authors received practical training from experts in systematic reviews and went through several practice sessions in order to collect the accurate data. Afterward, two authors individually examined the articles and a reviewer performed the final quality check. However, since two coders had to rely on their subjective judgment to evaluate each source, there were chances of errors. To reduce such errors, the authors decided to conduct interobserver agreement. Twenty randomly selected articles were first given to the two coders, and later, a cross-tabulation analysis was carried out to create a merged dataset. However, in cases where the two coders differed in their perspectives, a third party was involved to resolve the differences between them. The Kappa coefficient was 0.962, which implies an “almost perfect” strength of agreement [30].

**Table 2:** Suicide reporting guidelines

	Items
Avoid	1. Sensational coverage/headline 2. Overstatement/over-identification/over-simplification 3. Direct wording (e.g., suicide) 4. Mention of the method used 5. Details about the site/location 6. Use of photographs, video, social media links related to the act 7. Contents of a suicide note 8. Generalizing it as a constructive solution or as a coping mechanism 9. Speculating or jumping to conclusions 10. Romanticizing the act 11. Interviews with the bereaved
Recommended	12. Providing accurate information about where to seek help including hotline or local crisis phone numbers 13. Educating the public about the facts of suicide and suicide prevention

## Results

From 368 cases, the number of suicide reports was the highest in 2014 (38.9%), but overall, the number decreased from 143 cases in 2014 to 65 cases in 2017. In general, most of the suicide coverage from the five mainstream Korean newspapers adhered well to guidelines 1 (sensational coverage), 2 (overstatement), 6 (use of visual contents), 8 (generalization), 9 (speculation), 10 (romanticization), and 11 (interview). However, four items appeared to be incompliant with the guidelines and frequently appeared in reports of suicide. Direct wording was present in 63.9% of the total cases. Mentioning the method used and providing details about the site and location were high, at 68.2 and 74.5%, respectively. As many as 44.6% of the articles included the contents of the suicide note. On the recommended side, both guidelines 12 (inclusion of accurate information) and 13 (educating the public) were absent from nearly all suicide reports, and respectively only 1.4 and 2.2% of the reports included such information.

### Sensational coverage/headline

It is necessary for headline writers to think carefully about the content and its potential impact. The word “suicide” should not be used in the headline, and there should not be references to the method and site. In addition, language that sensationalizes suicide should be avoided [18, 20]. In this study, 23.9% (88 cases) of suicide reports intentionally capitalized on or emphasized the suicidal frequency, the sensational aspect of the suicide notes, or the motives for suicide in their headlines.

### Overstatement/over-identification/over-simplification

Over-simplification can be misleading and is unlikely to accurately reflect the complexity of suicide [21]. It is important not to minimalize the complex realities of suicide and the devastating impact on the people left behind [19]. In this study, 23.1% of the cases were shown to have committed the error of over-simplification. For example, some articles explained a K-pop star’s suicide in the context of the harsh idol-making system in South Korea, disregarding any other personal or social factors [31]. Similarly, there were articles that overstated the reason for the high suicide rate among Korean people in their 20s as being solely owing to the dismal unemployment condition [32].

### Direct wording

The word “suicide” should be avoided in the actual content of the article [18, 19]. Rather than using the word “suicide,” employing an alternative term, such as “death,” “passed away,” or “deceased,” is recommended. However, more than half of the reports (63.9%) directly used the word “suicide,” showing that this particular guideline is not well followed.

### Mention of the method used

An overly descriptive portrayal of the suicide may compel a vulnerable person to copy the act [21]. Even though this factor holds great risk to potential attempters, 68.2% of media suicide reports explained suicide methods in too much detail. For example, when the Songpa family suicide tragedy^1^ occurred in 2014, most readers were aware of the suicide method (e.g., burning charcoal).

### Details about the site/location

Certain locations are frequently chosen for attempting suicide [19]. The media should take particular care not to promote these suicide sites. However, contrary to the guidelines, 74.5% of the reports provided details about the site/location. This is especially dangerous in that it can increase the chances of such places becoming common sites for suicide. For example, high balconies and large bridges have become common places for people to commit suicide.

### Use of photographs, video, and social media links related to the act

Photographs, video footage, or social media links describing the scene of a suicide should not be used. In addition, great caution is required in using pictures of the deceased [18]. These images should not be prominently placed or glamorize the individual or act [19]. Approximately 12.5% of the printed newspapers utilized visual contents to attract more readers and some posted photographs of the actual sites where poisoning or self-burning occurred.

### Contents of a suicide note

Final text messages, social media posts, emails, and suicide notes from the deceased individual should not be published [21]. According to our results, 44.6% of the articles included contents of the suicide note. Some utilized the sensationalized part of the suicide note, such as “I should have killed more people before I committed suicide...” or “I couldn’t speak a single word...” [33, 34] so that they could attract more readers.

### Generalizing suicide as a constructive solution to the problem or as a coping mechanism

Language that misinforms the public about suicide, normalizes suicide or provides simple explanations for a suicide should be avoided [19]. This type of language may serve to desensitize the public to the gravity of suicide or even imply that death is a desirable outcome [21]. While a very high percentage of suicide reports (91.3%) avoided the generalization error, some authors generalized the suicide of a Lotte Group^2^ vice president as an act to save the company [35].

### Speculating or jumping to conclusions

Speculation about a death or the circumstances surrounding a death can easily be misreported or wrongly repeated as fact [19]. Hence, speculation about suicide, even if provided by a close family member, should be avoided. In the analysis, 36.7% of the articles were found to contain speculative statements. Some writers speculated on the reason for a suicide based solely on interviews with neighbors or acquaintances or even without any specific evidence.

### Romanticizing the act

Over-emphasizing community expressions of grief, such as “mourning,” may suggest that people are honoring the suicidal behavior rather than mourning the death [20]. Reporting suicide as a tragic waste and an avoidable loss is more beneficial in preventing further deaths [21]. Approximately 3.8% of the articles romanticized the act. In particular, when the vice principal of Danwon High School^3^ committed suicide out of guilt, some romanticized this as a result of his having felt accountable. Similarly, there were many articles expressing an extreme degree of mourning, respect, and sorrow when Robin Williams passed away [36].

### Interviews with the bereaved

Interviews may cause further pain to those who have survived the suicide attempt or to their family members [21]. Journalists, who are obsessed with publicizing a dramatic story, often take away family’s privacy and time [20]. When interviews take place, reporters should verify any information regarding the suicide since the words of the bereaved might be affected by their extreme emotions [21]. According to our study, there were 54 (14.7%) cases that mentioned interviews with the bereaved family. In many circumstances, these were interviews with the wife, husband, or parents of the suicide victim.

### Providing accurate information about where to seek help including hotline or local crisis phone numbers

Since suicide coverage may impel readers to take similar action, the articles should always end with instructions on how to seek help [20]. The information must be accurate, accessible, and concise for it to be helpful [21]. Surprisingly, there were only five newspaper articles (1.4%) that provided accurate information on how to seek help. In other words, most of the articles were only focused on delivering information on the suicidal act and left out preventative information.

### Educating the public about the facts of suicide and suicide prevention

Since there are many misconceptions about suicide, which the public tends to accept it as facts, it is necessary to provide factual information about suicide [20]. In addition to carefully researching the facts about suicide, it is always helpful to report on suicide prevention [21]. Only eight articles (2.2%) included factual information about suicide and its possible consequences. This again shows how ignorant and passive South Korean newspapers have been in preventing suicide (Tables 3 and 4).

**Table 3:** Number of Suicide Reports

Year	Number of suicide reports, N (%)
2014	143 (38.9)
2015	78 (21.2)
2016	82 (22.3)
2017	65 (17.7)

**Table 4:** Number of Articles for Each Guideline Item

Items	Presence, N (%)	Absence, N (%)
1. Sensational coverage/headline	88 (23.9)	280 (76.1)
2. Overstatement/over-identification/over-simplification	85 (23.1)	283 (76.9)
3. Direct wording (e.g., suicide)	235 (63.9)	133 (36.1)
4. Mention of the method used	251 (68.2)	117 (31.8)
5. Details about the site/location	274 (74.5)	94 (25.5)
6. Use of photographs, video, and social media links related to the act	46 (12.5)	322 (87.5)
7. Contents of a suicide note	164 (44.6)	204 (55.4)
8. Generalizing it as a constructive solution to the problem or as a coping mechanism	32 (8.7)	336 (91.3)
9. Speculating or jumping to conclusions	135 (36.7)	233 (63.3)
10. Romanticizing the act	14 (3.8)	354 (96.2)
11. Interviews with the bereaved	54 (14.7)	314 (85.3)
12. Providing accurate information about where to seek help including hotline or local crisis phone numbers	5 (1.4)	363 (98.6)
13. Educating the public about the facts of suicide and suicide prevention	8 (2.2)	360 (97.8)

## Discussion

This study explored how effective five Korean newspapers are at following the country’s suicide reporting guidelines. Owing to the exposure of high risk in suicide, strengthening the suicide reporting guidelines is essential in South Korea. Our findings on using direct wording (63.9%), providing detailed information (74.5%), and reporting the contents of the suicide note (44.6%) were very similar to those of Kim’s [27] study, where percentage of these items were 64, 75 and 45%, respectively. The overall percentage of articles speculating or jumping to conclusions (36.7%) was similar to the results of Creed and Whitley (34.8%) [23] and Utterson et al.’s (30.1%) [26] studies. Similarly, the results for providing hotline information (1.4%) and educating the public (2.2%) aligned with the studies by Ramadas and Kuttichira [24] and Harshe et al. [25], who found nearly zero cases for these guideline items. Contrary to other international studies that showed less than 30% of discordance in providing extensive details about the method, site/location, and suicide note, our findings showed much higher percentages of discordance with these items—68.2, 74.5 and 44.6%, respectively—in South Korea [16, 25, 26, 37].

More than half of the articles included direct wording (63.9%), made mention of the location (74.5%), and made mention of the method used (68.2%). This is especially problematic in that it provides information for potential attempters to execute the act [38]. The public is already familiar with popular suicide methods and locations such as burning charcoal in closed rooms or jumping off a bridge over the Han River in Seoul, Korea [27]. Moreover, special attention should be paid to the adolescent population. According to Phillips and Carstensen [39], the impact of suicide stories on subsequently completed suicides have been reported to be the greatest among teenagers. The chances of adolescents committing copycat suicides are high, as their self-identities are not yet fully developed; this allows them to be easily influenced by celebrities [40]. Additionally, the youth are likely to be influenced by newspaper stories that are more prominent (e.g., front-page placement or inclusion of a picture), more explicit (e.g., with headlines containing the word “suicide” or specifying the method used), or more detailed (e.g., including the name of the deceased, the details of the method, or a suicide note) [41].

Another critical issue lies on having so few articles with help-seeking information or factual information about suicide. The need to provide appropriate suicide information becomes a significant task. Additionally, when leaving out such information, suicide becomes the problem of one individual, rather than a social or general public health problem of which we must all be aware [38].

Moreover, the results of this study provide important insights for examining the weaknesses in the existing Korean suicide reporting guidelines. Even though visual contents and suicide notes are proven to be influential factors for the actual suicide attempt [18, 21, 41], references to those items were left out of Suicide Reporting Guideline 2.0. In the study by Ramadas and Kuttichira [24], 37% of cases (*N* = 3270) included photographs of the deceased, the method, and the scene. Harshe et al. [25] separated the visual contents section into three specific guideline items. Out of 708 cases, 20.29% had “a photograph of the victim,” 11.98% had “a photograph of the location” and 14.42% had “a graphical illustration to depict the suicide/location,” meaning that in total, 46.69% included some type of visual contents within the article. In an analysis of Chinese newspapers, 57.5% of articles had photos and 4.7% had graphics; in other words, 62.2% had visual content items [42]. Having to confirm the prevalence and importance of this particular guideline item, those covering visual contents or suicide notes should be included in the updated version of the Korean suicide reporting guidelines. However, most importantly, media as a primary stakeholder should engage more proactively with these guideline items.

Currently, there are three organizations that monitor suicide coverage in the media: the Korea Suicide Prevention Center, the Korea Communications Standards Commission, and the Journalists Association of Korea [43, 44]. The Korea Suicide Prevention Center [44] has established the Suicide Prevention Committee with various stakeholders to broaden the monitoring area, educate the public, publish the monitoring guide, and raise public awareness on the issue of suicide prevention. Despite the importance of this role, a lack of staff and budget limit the organization’s scope. According to the Korea Communications Standards Commission [43], suicide coverage with too much detail, romanticization or justification, and speculation is prohibited in broadcasting. However, there were too many instances whereby this clause was violated thus Korea Communications Standards Commission has announced a desire to strengthen the monitoring regulations [43]. However, these agencies generally lack the structure and power to effectively manage suicide prevention on a nationwide scale. Considering the gravity of this issue, the Korean government should reinforce the monitoring system and implement a stricter editorial policy to effectively counteract against the reckless suicide coverages released on media.

For the establishment of an efficient monitoring system, we may refer to the National Strategy for Suicide Prevention proposed by the Government of the United Kingdom. The Government of the United Kingdom made a collective effort in suicide prevention by setting up several goals and forming an alliance system that are “supporting the media in delivering sensitive approaches to suicide and suicidal behavior,” “supporting research, data collection, and monitoring,” and creating “the National Suicide Prevention Alliance” [45]. Under the current administration, Korea has recently announced a 5-year “National Suicide Prevention Action Plan (2018-22)” aiming to reduce the high suicide rate in the country. According to the Korean Ministry of Health and Welfare [46], the action plan has four main pillars: 1) providing a basis for a suicide prevention policy, 2) conducting a high-risk suicide population assessment, 3) establishing active intervention management for the high-risk population, and 4) providing follow-up service and support for suicide attempters. However, creating stronger regulations for suicide reporting guidelines and monitoring suicide coverage in the media has not been included in this plan. The results of our study may provide a basis and be a helpful resource for the establishment of stronger regulations for suicide reporting guidelines as well as a structured national surveillance system on suicide coverage in the media.

Although this study did not cover online suicide reporting, the number of online articles outweighs the number of printed articles; therefore, the problem with suicide coverage is expected to be greater when online articles are taken into account. This is because online journalists who compete with one another for more clicks and views tend to use sensational headlines and content to attract readers [47]. Reporting about suicide is especially problematic in that many focus on highlighting the issue as something shocking and gossip-worthy; nothing more [17, 47]. As such, an online regulation policy and penalty system (when too many details or overly sensational information are given) should also be considered as part of a suicide prevention policy.

Our study provides an important rationale as to why there should be stronger regulations for suicide reporting guidelines. However, it has some limitations. First, our sample was limited to “printed” articles that were available online. We could have missed articles that were not yet uploaded or available online. Second, choosing only five mainstream Korean newspapers for the analysis might have influenced the overall results of the study. Despite these limitations, this was the first study to be conducted after the revision of the Korean guidelines in 2013; thus, our findings will be a useful source for further studies of this kind.

## Conclusions

This study is notable in that South Korea currently lacks studies on suicide reporting guidelines. From our study, it became clear that the current guidelines are ineffective. To reduce suicide rates and attempts and to yield more positive outcomes for the future, our study strongly suggests avoiding the word “suicide” and detailed descriptions, but rather providing factual yet helpful information to those who are considering suicide. We expect our findings to be utilized in many suicide-related studies and to contribute to the establishment of more effective and concrete suicide reporting guidelines as well as monitoring systems.

## Abbreviations

AFSP: American Foundation for Suicide Prevention
CDC: Centers for Disease Control and Prevention
NSPA: National Suicide Prevention Alliance
OECD: Organisation for Economic Co-operation and Development
WHO: World Health Organization

## Footnotes

1 The Songpa family suicide tragedy: A family consisting of two daughters and their mother committed suicide on February 26, 2014. Even though they were experiencing hardship (chronic illness, economic difficulty), there was no proper social safety net to help them recover. This event became the trigger point to reach out to vulnerable groups located within the blind spot of the Korean social welfare system.

2 According to the Korea Fair Trade Commission report in 2017, the Lotte Group is the fifth largest corporate in South Korea in terms of its total assets.

3 Danwon High School: The school involved in the Sewol Ferry disaster. The Sewol Ferry sank off Jindo island, South Korea, on 16 April 2014, killing 304 people, almost all of them Danwon high school students.
